# Obstetric Life Support Education for Maternal Cardiac Arrest: A Randomized Clinical Trial

**DOI:** 10.1001/jamanetworkopen.2024.45295

**Published:** 2024-11-15

**Authors:** Andrea D. Shields, Jacqueline Vidosh, Charles Minard, Brook Thomson, Kristen Annis-Brayne, Makayla Murphy, Laurie Kavanagh, Cheryl K. Roth, Monica A. Lutgendorf, Meredith L. Birsner, Stephen J. Rahm, Les R. Becker, Vincent Mosesso, Brian Schaeffer, Matthew Streitz, Utpal Bhalala, Andrea Gresens, John Phelps, Benjamin Sutton, Richard Wagner, Lissa M. Melvin, Kathleen Zacherl, Laura Karwoski, James Behme, Alex Hoeger, Peter E. Nielsen

**Affiliations:** 1 Department of Obstetrics and Gynecology, University of Connecticut Health, Farmington; 2 Department of Obstetrics and Gynecology, San Antonio Uniformed Services Health Education Consortium, San Antonio, Texas; 3 Institute for Clinical and Translational Research, Baylor College of Medicine, Houston, Texas; 4 Department of Obstetrics and Gynecology, The University of Texas Health Science Center at San Antonio, San Antonio; 5 Department of Rehabilitation Medicine, University of Washington, Seattle; 6 Department of Obstetrics and Gynecology, HonorHealth Shea Medical Center, Scottsdale, Arizona; 7 Department of Obstetrics and Gynecology, Uniformed Services University of the Health Sciences, Bethesda, Maryland; 8 Department of Obstetrics and Gynecology, St Luke’s University Health Network, Bethlehem, Pennsylvania; 9 Centre for Emergency Health Sciences, Spring Branch, Texas; 10 MedStar Institute for Innovation, Simulation Training, and Education Lab, MedStar Health, Washington, DC; 11 Department of Emergency Medicine, University of Pittsburgh School of Medicine, Pittsburgh, Pennsylvania; 12 Spokane Fire Department, Spokane, Washington; 13 Department of Emergency Medicine, San Antonio Uniformed Services Health Education Consortium, San Antonio, Texas; 14 Department of Pediatrics, Amistad Health, Corpus Christi and Texas A&M University, College Station; 15 Department of Emergency Medicine, Methodist Healthcare, San Antonio, Texas; 16 Department of Business, Our Lady of the Lake University, San Antonio, Texas; 17 Department of Obstetrics & Gynecology, Christus Children’s/Baylor College of Medicine, San Antonio, Texas; 18 Health Information Technology Department, University of Connecticut Health, Farmington; 19 Simulation Center, University of Connecticut Health, Farmington

## Abstract

**Importance:**

Management of maternal cardiac arrest (MCA) requires understanding the unique physiology of pregnancy and modifications to life support. Health care professionals have historically demonstrated inadequate knowledge and skills necessary to treat MCA.

**Objective:**

To evaluate the effect of Obstetric Life Support (OBLS) education on health care professionals’ cognitive performance, skills, and self-efficacy in managing MCA.

**Design, Setting, and Participants:**

In this single-masked randomized clinical trial, 46 health care professionals, including emergency medical service and hospital staff representing diverse specialties, were randomized to intervention or control groups at a single academic medical center in Farmington, Connecticut between May 1, 2022, and July 23, 2023.

**Intervention:**

The intervention group received OBLS education, which included a blended learning curriculum with simulation-based training on common maternal medical emergencies that lead to MCA. Participants were assessed for knowledge, confidence, and skills (eg, megacode scores as team leaders during MCA simulations). Intervention participants were compared with control group participants who received no OBLS education.

**Main Outcomes and Measures:**

The primary outcome was cognitive scores. Secondary outcomes included megacode scores rated by experienced OBLS instructors masked to assignment groups, combined assessment pass rates, and cognitive and confidence scores at baseline and 6 and 12 months after education. Data were analyzed from January 2024 to May 2024.

**Results:**

Forty-six participants (mean [SD] age, 41.1 [16.2] years; 24 [52%] women) were randomized. Despite most participants holding certification in basic and advanced cardiac life support, significant between-group differences were identified in knowledge, skills, and confidence. Mean (SD) cognitive scores were 79.5% (9.4%) in the intervention group vs 63.4% (12.3%) in the control group (*P* < .001). Mean (SD) megacode skills were higher in the intervention vs control group (91.0% [5.0%] vs 61.0% [12.0%], *P* < .001), as were confidence scores (72.7 [13.3] vs 56.2 [17.9] points, *P* = .002). Combined assessment pass rates were 90% in the intervention group compared with 10% in the control group (*P* < .001).

**Conclusions and Relevance:**

In this randomized clinical trial, OBLS education significantly improved health care professionals’ knowledge, skills, and confidence in managing MCA. These findings underscore the urgent need for implementation of a standardized MCA curriculum nationwide, especially as the US continues to face unacceptably high maternal mortality rates.

**Trial Registration:**

ClinicalTrials.gov Identifier: NCT05355519

## Key Points

**Question:**

Does a simulation-based blended learning intervention improve participant knowledge, skills, and confidence in managing maternal cardiac arrest (MCA)?

**Findings:**

In this randomized clinical trial of 46 emergency medical service and hospital-based health care professionals from various specialties, a simulation-based blended learning intervention significantly improved cognitive performance, self-efficacy, and resuscitation skills when managing an MCA simulation assessed by masked instructors.

**Meaning:**

A simulation-based blended learning curriculum is effective in teaching the management of maternal medical emergencies and MCA, a known deficiency among health care professionals that contributes to severe maternal morbidity and mortality and health care disparities.

## Teaser

This randomized clinical trial of emergency medical service and hospital-based health care professionals examines whether a simulation-based blended learning intervention improves participant knowledge, skills, and confidence in managing maternal cardiac arrest.

## Introduction

Maternal cardiac arrest (MCA) is a rare event, with an incidence of 1 in 3885 during admission for delivery.^1^ In 2015, the American Heart Association published its Scientific Statement on Cardiac Arrest in Pregnancy,^2^ recommending implementation of evidence-based practices for MCA response^3,4^ and emphasizing specialized interventions during resuscitation. However, despite the US leading developed nations in the maternal mortality ratio at 22.3 maternal deaths per 100 000 live births^5^ and these existing evidence-based recommendations for MCA response and algorithms designed to assist health care professionals in optimizing the management of MCA,^3,6,7,8^ there are no specific MCA training or credentialing requirements. In contrast, the pediatric-related mortality ratio for pediatric cardiac arrest is lower at 15.3 deaths per 100 000 (for those aged 5-14 years),^9^ with pediatric-specific training including Basic Life Support (BLS) for infants and children and Pediatric Advanced Life Support (PALS), typically required for both in-hospital and prehospital clinicians.

Significant gaps in MCA knowledge and skills also exist among first responders. The current prehospital incidence of MCA is estimated at 1.85 per 100 000 population, with a 16% maternal survival rate.^10^ However, prehospital survival rates improve significantly when the chain of survival (eg, early recognition and activation of emergency response system, early cardiopulmonary resuscitation, rapid defibrillation, and advanced life support) is optimized.^11^ The observed low prehospital MCA survival rate may reflect suboptimal bystander cardiopulmonary resuscitation response, emergency medical systems (EMS) response time, EMS physician nonattendance, and a lack of EMS training on critical modifications to BLS and advanced cardiac life support (ACLS) in MCA.^12^ The increasing number of prehospital births, now at their highest levels since 1990, further underscores the urgent need for enhanced prehospital personnel training in effective MCA management.^13^

Current training tools for MCA are insufficient and lack performance assessment features. Obstetric Life Support (OBLS) is a comprehensive training program designed to address this gap by equipping health care professionals with the knowledge and skills to manage MCA effectively, including performance assessments for individuals and teams. This study evaluates the effect of OBLS training on cognitive performance, self-efficacy, megacode evaluations (observations of participant technical resuscitation and behavioral team leadership skills during an MCA scenario), and combined assessment pass rates among health care professionals in prehospital and in-hospital environments.

## Methods

### Trial Design and Oversight

We conducted a single-masked randomized clinical trial from May 1, 2022, through July 23, 2023, at a single site in Farmington, Connecticut. The trial protocol and statistical analysis plan are available in Supplement 1 and follow the Consolidated Standards of Reporting Trials (CONSORT) reporting guidelines for randomized trials. Site investigators (A.D.S. and C.M.) designed and supervised the conduct of the trial.

All participating OBLS instructors were masked to the group assignment. Data collection was performed by the research coordinators (L. Kavanagh, K.A.-B., M.M.), and the biostatistician (C.M.) performed the data analyses. All authors interpreted the data and ensured the accuracy and completeness of the data and the trial’s adherence to the protocol. The trial was conducted following Good Clinical Practice guidelines and the principles of the Declaration of Helsinki.^14^ The University of Connecticut Health institutional review board (IRB) reviewed the study to help ensure that the rights and welfare of the research participants were protected and that the research study was carried out in an ethical manner. The trial protocol received an exempt determination with a waiver of consent because the trial was deemed minimal risk due to normal educational practices. Verbal informed assent was obtained prior to initiating any trial-related procedures as data were being collected from participants.

### Participants

Study participants were English-speaking health care professionals (aged >18 years) who work in prehospital or in-hospital environments and care for reproductive-aged women. For prehospital OBLS education, teams had a maximum of 4 participants per class and consisted of 2 crews, one responding after the other, of EMS health care professionals, including paramedics, emergency medical technicians, law enforcement, and firefighters. For in-hospital OBLS education, teams had a maximum of 6 participants consisting of at least 1 health care professional from the emergency department, family medicine, intensive care unit, or surgery (obstetrics); 1 anesthesiologist; 1 graduate medical education trainee; and 1 nurse from the emergency department, family medicine, intensive care unit, or obstetrics or labor and delivery. For the prehospital and in-hospital groups, at least 1 health care professional on each team had 5 years of experience. Exclusion criteria included anyone who participated in the OBLS pilot validation trial or did not assent to be randomized.

### Trial Procedures and End Points

Participants who fulfilled randomization criteria were randomly assigned in a 1:1 ratio to receive OBLS education or no education. Randomization was performed using an interactive web response system in a stratified, permuted block design. Randomization was stratified by hospital status (in-hospital vs prehospital). One study personnel (L. Kavanagh) enrolled and randomly assigned participants to groups.

**Figure 1.**
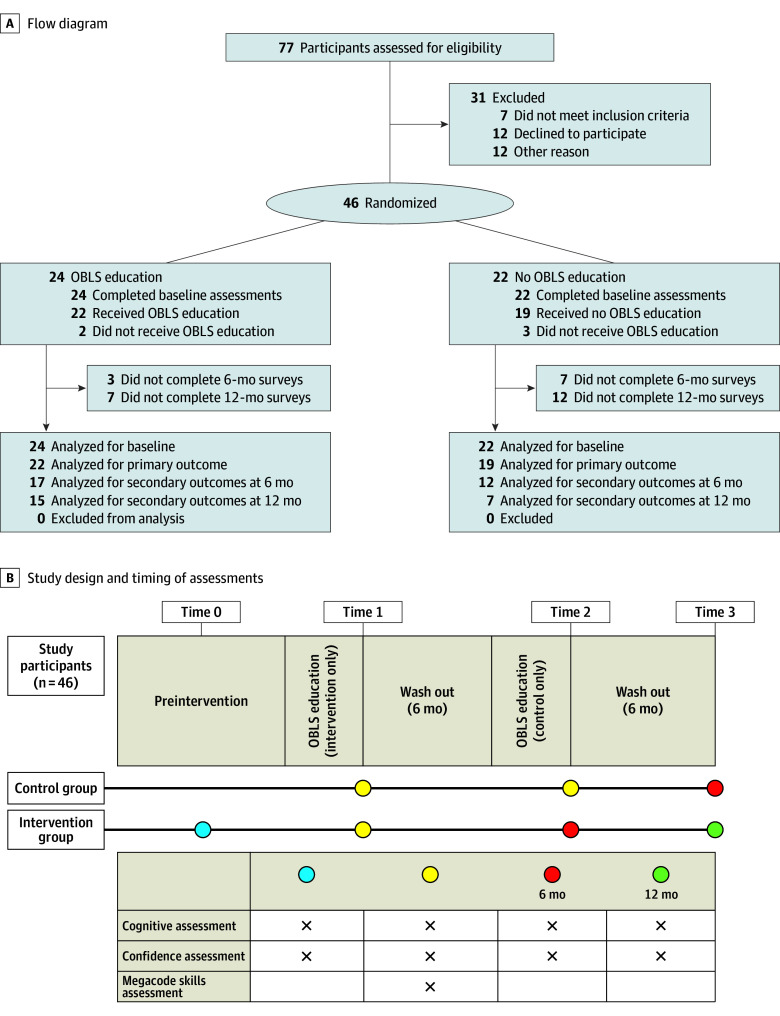
CONSORT Flow Diagram and Study Design and Timing of Assessments OBLS indicates Obstetric Life Support.

Figure 1 summarizes the overall study design. Participants randomized to the intervention arm received cognitive and confidence evaluations at enrollment (time 0), after intervention (time 1), 6 months after enrollment (time 2), and 12 months after enrollment (time 3). Megacode evaluations (eFigures 1-3 in Supplement 2) were completed during time 1.

Participants assigned to the control arm received cognitive and confidence evaluations at enrollment (time 1) but did not receive the educational intervention until 6 months after enrollment (time 2). Cognitive and confidence evaluations were then assessed after the intervention at their 6-month time point (time 3). There were no changes to the methods after the trial started.

### Intervention

The OBLS is a comprehensive, interdisciplinary, simulation-based training curriculum on MCA prevention and treatment based on the accepted evidence-based guidelines from the American Heart Association.^15^ The intervention is described in detail in Supplement 1. Participants were evaluated through baseline (cognitive and confidence) and postcourse assessments (cognitive, confidence, and megacode) as well as 6- and 12-month assessments of cognitive performance and confidence. To pass the course, participants needed to achieve a passing score of 70% or higher or 67% or higher on the postcourse cognitive examination for in-hospital and prehospital training, respectively, and greater than 74% on the megacode assessments. Cut scores for assessment were established by an expert panel through an Angoff process as previously described.^15^ The OBLS instructors participating in megacode evaluations were masked to the participant assignment. To ensure masking and consistency, a single site investigator not participating in megacode evaluations (A.D.S.) oriented control teams to the simulation environment and equipment using a standardized checklist (eFigure 4 in Supplement 2) before megacode testing commenced. Each control participant was then assessed as a team leader during a simulated megacode of an MCA.

### Data Collection

Demographic and baseline variables included age (years), gender identity by self-selection (woman, man, or nonbinary), and race and ethnicity by self-selection (Asian, Black or African American, Hispanic or Latinx, White, and other). The “other” race and ethnicity category included participants who answered “prefer not to respond.” Additional baseline variables included number of prior simulation exercises (<5, 5-10, >10 and ≤20, and >20), prior experience as a simulation instructor (yes or no), and certification status (current, previous, or never) in BLS, ACLS, Advanced Trauma Life Support, Prehospital Trauma Life Support (PHTLS), Neonatal Resuscitation Program, and BLS or ACLS instructor.

### Outcomes

The primary outcome of interest was the cognitive score during time 1. During this time, the effectiveness of the OBLS education was assessed in the intervention arm vs the control arm. Secondary end points included megacode and self-efficacy scores at time 1 and cognitive and self-efficacy scores at other times.

Cognitive assessments were developed and validated for online administration for participants from in-hospital and prehospital environments.^15^ Prehospital participants were further assigned to a basic vs advanced cognitive assessment based on their roles (eg, emergency medical technicians who cannot administer medications or use advanced airways received the prehospital basic assessment). Each test item was equally weighted, and a final score was tabulated by computing the percentage of items correct. Items with missing responses were counted as incorrect.

Megacode checklists were previously developed and validated, and a cut score was determined using a modified Angoff method.^15^ Masked, trained evaluators used the checklist to assess team leader performance during a megacode MCA scenario. Checklist domains included preresuscitation, resuscitation, post–return of spontaneous circulation care, team leader performance, and communication and teamwork. The passing score was a mean score greater than 74% with the absence of critical failures, defined as no critical elements scored 0. Critical elements for in-hospital and prehospital assessments, previously identified by the expert panel,^15^ included timely recognition of cardiac arrest, initiation of high-quality chest compressions, effective ventilation, maintenance of left uterine displacement, defibrillation (when indicated), and completion of resuscitative cesarean delivery within 5 minutes at the site of arrest (in-hospital only). On the basis of their level of certification, participants from prehospital environments were further assigned to a basic or advanced megacode checklist assessment. Each test item was on a Likert scale, with a final score tabulated by adding item scores and converting to a 100-point scale, with 1 indicating lowest performance and 100 indicating perfect performance. Combined assessment pass rates were based on passing both the cognitive and megacode assessments based on expert-set cut scores.^15^

Online self-efficacy assessments were validated for 4 categories: clinical confidence, procedural confidence, knowledge confidence, and communication confidence. Each item was equally weighted, and a final score was tabulated by adding item scores and converting to a 100-point scale, with 1 indicating lowest performance and 100 indicating perfect performance.

### Statistical Analysis

We hypothesized that OBLS education would improve knowledge, megacode scores, and self-efficacy compared with usual training (control). The primary outcome of interest was cognitive test scores during time 1, and the primary hypothesis test compared mean cognitive test scores during time 1 between study arms using an independent, 2-sample *t* test. Preliminary data estimated the SD of cognitive test scores to be approximately 12 points.^15^ Because the cognitive test was developed specifically for this study, 10 points was designated as a clinically important difference because this is considered a standard difference between letter grades. A sample size of 24 participants per group (for a total of 48) provided 80% power to reject the null hypothesis that mean cognitive test scores were equal between study arms using an independent, 2-sample *t* test assuming a common SD of 12 and 2-sided α = .05.

Baseline demographics and professional characteristics were summarized by means (SDs) or numbers (percentages). Participant age at enrollment was computed as the integer age (rounded down) at the time of enrollment. Summary statistics were stratified by treatment arm and compared using independent, 2-sample *t* tests, Wilcoxon rank sum test, or χ^2^ test as appropriate.

Primary and secondary efficacy end points were analyzed according to the intention-to-treat principle, with analyses including all participants who had undergone randomization. Scores for missing assessments (cognitive, self-efficacy, and megacode) were not imputed. All available scores were included in the primary analysis of the full population. A subgroup analysis of the megacode assessment outcomes was performed based on the in-hospital vs prehospital subgroups.

The primary outcome of interest was cognitive test scores during time 1, computed as the percentage of correct answers, ranging from 0% to 100%. The primary hypothesis test compared mean cognitive scores between treatment arms during time 1 using an independent, 2-sample *t* test assuming homogenous variance as assessed at the 2-sided α = .05 level. Secondary analysis compared mean cognitive scores between treatment arms across all time points using a general linear mixed model. The model included fixed effects for arm, time (ie, times 0, 1, 2, and 3), and the arm × time interaction term. If the interaction term was significant at the α = .05 level, then the model assessed all pairwise comparisons using linear contrasts, and *P* values were adjusted for multiple comparisons using the Tukey method. A 2-sided *P* < .05 level was considered statistically significant. The model estimated means with 95% CIs by arm and time point. The matrix of correlated residuals assumed an unstructured format.

Secondary outcomes included self-efficacy, megacode scores, and combined assessment pass rates. Self-efficacy scores were computed as the sum of item responses converted to a 100-point scale. Scores ranged from 0 to 100 points, with 1 indicating lowest performance and 100 indicating perfect performance. Megacode scores were computed as the percentage of items correct and ranged from 0% to 100%. Mean (SD) scores of the megacode assessment were observed at a single time point and compared between treatment arms using the Wilcoxon rank sum test. Combined assessment pass rates were reported as the percentage of participants who passed using the χ^2^ test comparing pass rates between the 2 treatment arms. Statistical significance was assessed at α = .05 for all secondary outcomes. SAS software, version 9.4 (SAS Institute Inc) was used for statistical analysis. Data were analyzed from January 2024 to May 2024. Additional details regarding the statistical analysis are provided online (Supplement 1).

## Results

### Trial Participants

The trial included a total of 46 participants (mean [SD] age, 41.1 [16.2] years; 24 [52%] women, 20 [44%] men, 1 [2%] nonbinary, and 1 [2%] with missing data; 3 [7%] Asian, 3 [7%] Black or African American, 1 [2%] Hispanic or Latinx, 27 [58%] White, and 1 [2%] other [those who preferred not to respond]); 24 were assigned to the intervention arm and 22 to the control arm (Table 1). Although most participants had participated in more than 20 simulation exercises in their careers, only 37% indicated they had experience as a simulation instructor. Most participants were currently or previously certified in BLS (44 [95%]) and ACLS (32 [70%]), with some participants indicating current or previous certification as a BLS instructor (11 [24%]) or ACLS instructor (8 [18%]). Fewer were currently or previously certified in PALS (20 [43%]), Neonatal Resuscitation Program (17 [37%]), PHTLS (9 [20%]), or Advanced Trauma Life Support (6 [13%]). Thirty-one participants were excluded for not meeting inclusion criteria (n = 7), declining to participate (n = 12), or for other reasons (n = 12), such as failing to return telephone calls or emails (Figure 1). There was 1 randomization error that occurred during the trial: A prehospital-advanced participant was randomized to a prehospital-basic control arm but underwent prehospital-advanced cognitive and megacode assessments and remained in the prehospital group for analysis. There was no harm to the participants during this study.

**Table 1.** Baseline Demographics of the Study Participants

Characteristic	No. (%) of participants^a^		
	All (N = 46)	Control (n = 22)	Intervention (n = 24)
Age, mean (SD), y	41.1 (16.2)	45.6 (19.7)	36.9 (10.9)
Gender identity (if specified)			
Woman	24 (52)	13 (59)	11 (46)
Man	20 (44)	9 (41)	11 (46)
Nonbinary	1 (2)	0	1 (4)
Missing	1 (2)	0	1 (4)
Race and ethnicity			
Asian	3 (7)	1 (4)	2 (8)
Black or African American	3 (7)	2 (9)	1 (4)
Hispanic or Latinx	1 (2)	1 (5)	0
White	27 (58)	13 (59)	14 (58)
Other^b^	1 (2)	0	1 (4)
Missing	11 (31)	5 (29)	6 (33)
No. of times participating in a simulation exercise			
≤5	7 (15)	2 (9)	5 (21)
>5 and ≤10	4 (9)	1 (5)	3 (13)
>10 and ≤20	13 (28)	6 (27)	7 (29)
>20	22 (48)	13 (59)	9 (38)
Missing	0	0	0
Experience as a simulation instructor			
No	28 (61)	13 (59)	15 (63)
Yes	17 (37)	9 (41)	8 (33)
Missing	1 (2)	0	1 (4)
ACLS			
Currently certified	23 (50)	10 (45)	13 (54)
Never certified	14 (30)	8 (36)	6 (25)
Previously certified	9 (20)	4 (18)	5 (21)
Missing	0	0	0
ACLS instructor			
Currently certified	4 (9)	2 (9)	2 (8)
Never certified	38 (83)	19 (86)	19 (79)
Previously certified	4 (9)	1 (5)	3 (13)
Missing	0	0	0
BLS			
Currently certified	37 (80)	17 (77)	20 (83)
Never certified	2 (4)	2 (9)	0
Previously certified	7 (15)	3 (14)	4 (17)
Missing	0	0	0
BLS instructor			
Currently certified	7 (15)	3 (14)	4 (17)
Never certified	35 (76)	19 (86)	16 (67)
Previously certified	4 (9)	0	4 (17)
Missing	0	0	0
PALS			
Currently certified	18 (39)	7 (32)	11 (46)
Never certified	26 (57)	13 (59)	13 (54)
Previously certified	2 (4)	2 (9)	0
Missing	0	0	0
NRP			
Currently certified	11 (24)	7 (32)	4 (17)
Never certified	29 (63)	14 (64)	15 (63)
Previously certified	6 (13)	1 (5)	5 (21)
Missing	0	0	0
ATLS			
Currently certified	2 (4)	2 (9)	0
Never certified	39 (85)	20 (91)	19 (79)
Previously certified	4 (9)	0	4 (17)
Missing	1 (2)	0	1 (4)
PHTLS			
Currently certified	6 (13)	3 (14)	3 (13)
Never certified	30 (65)	15 (68)	15 (63)
Previously certified	3 (7)	1 (5)	2 (8)
Missing	7 (18)	3 (16)	4 (20)

Abbreviations: ACLS, Advanced Cardiac Life Support; ATLS, Advanced Trauma Life Support; BLS, Basic Life Support; NRP, Neonatal Resuscitation Program; PALS, Pediatric Advanced Life Support; PHTLS, Prehospital Trauma Life Support.

^a^ Unless otherwise indicated.

^b^ “Other” category includes participants who answered “prefer not to respond.”

Baseline characteristics were similar in the 2 groups, except the control group had more participants currently certified in Advanced Trauma Life Support (2 [9%] vs 0, *P* = .049) (Table 1). There were no significant differences in age, gender identity, race or ethnicity, or prior experience with simulation exercises. There were also no significant differences between the control and intervention groups in current certification in BLS (17 [77%] vs 20 [83%], *P* = .32) or ACLS (10 [45%] vs 13 [54%], *P* = .70), PALS (7 [32%] vs 11 [46%], *P* = .25), and PHTLS (3 [14%] vs 3 [13%], *P* = .86). There was no difference in experience as a simulation instructor (9 [41%] vs 8 [33%], *P* = .67), ACLS instructor (2 [9%] vs 2 [8%], *P* = .63), or BLS instructor (3 [14%] vs 4 [17%], *P* = .11) between the control and intervention groups.

**Figure 2.**
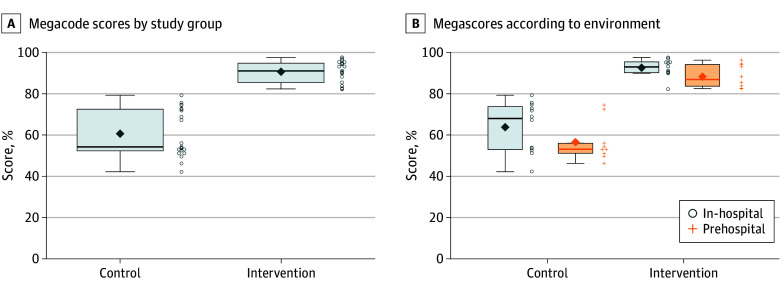
Megacode Scores According to Study Group and Environment Each test item was on a Likert scale, with a final score tabulated by adding item scores and converting to a 100-point scale, with 1 indicating lowest performance and 100 indicating highest performance. The ends of the boxes represent the 25th and 75th percentiles; horizontal line inside the box, the mean; whiskers, the upper and lower adjacent values; diamond, mean; open circles and plus signs, points that fall beyond the whiskers.

Table 2 summarizes the results for time 1. Mean (SD) cognitive scores were higher in the intervention group than the control group (79.5% [9.4%] vs 63.4% [12.3%], *P* < .001). Mean megacode scores were higher in the intervention group than the control group (91.0% [5.0%] vs 61.0% [12.0%], *P* < .001). This finding was also observed when stratified by environment (prehospital vs in-hospital) (Figure 2). Additionally, the combined assessment pass rates were markedly higher in the intervention group compared with the control group (91% vs 10%, *P* < .001). Mean (SD) confidence scores were higher in the intervention group than the control group (72.7 [13.3] vs 56.2 [17.9] points, *P* = .002).

**Table 2.** Summary Statistics of Cognitive, Megacode, and Confidence Scores and Combined Assessment Pass Rates Between Intervention and Controls During Time 1

Outcome measure	Summary statistic		*P* value
	Intervention (n = 24)	Control (n = 22)	
Cognitive score, mean (SD)	79.5 (9.4)^a^	63.4 (12.3)^a^	<.001^b^
Megacode score, mean (SD)	91.0 (5.0)^a^	61.0 (12.0)^c^	<.001^d^
Confidence score, mean (SD)	72.7 (13.3)^e^	56.2 (17.9)^a^	.002^b^
Combined assessment pass rate, %	91^a^	10^c^	<.001^f^

^a^ Data are missing for 3 participants.

^b^ Independent, 2-sample *t* test.

^c^ Data are missing for 1 participant.

^d^ Wilcoxon rank sum test.

^e^ Data are missing for 4 participants.

^f^ χ^2^ Test.

**Figure 3.**
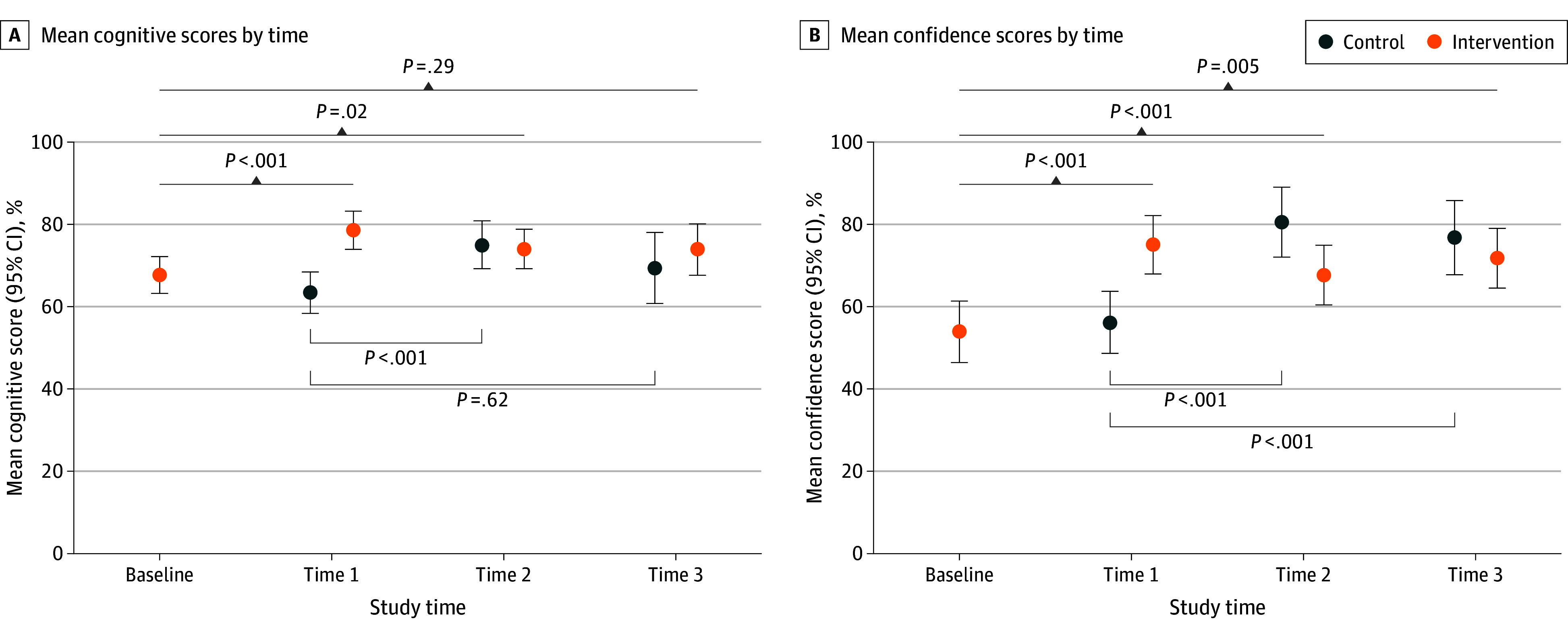
Mean Cognitive and Confidence Scores by Study Arm and Time Each item was equally weighted, and a final score was tabulated by adding item scores and converting to a 100-point scale, with 1 indicating no confidence and 100 indicating total confidence. Error bars indicate 95% CIs.

When analyzing within-group differences, during time 1, the intervention group achieved improvements in post-OBLS education cognitive (10.8 points; *P* < .001) and confidence (21.1 points; *P* < .001) results compared with their baseline. The improvement in confidence persisted at the 12-month assessment (Figure 3). Improvements in cognitive results persisted at the 6-month assessment (6.3 points; *P* = .01) but were not significant by 12 months (6.2 points; *P* = .29). Compared with their baseline, the control group posted higher post-OBLS education cognitive scores (11.6 points; *P* < .001), but this difference did not remain significant at 6 months (6.0 points; *P* = .62) (Figure 3). Control group confidence scores remained higher than baseline after OBLS education (24.3 points; *P* < .001) and at 6 months (20.6 points; *P* < .001).

## Discussion

This study demonstrates the effectiveness of the OBLS program in improving health care professionals’ ability to manage a simulated MCA event. Compared with controls, participants who completed OBLS education had higher cognitive performance, self-efficacy, ability to lead an MCA megacode, and combined assessment pass rates. These improvements were seen in both in-hospital and prehospital health care professionals, highlighting the program’s broad applicability across different health care settings. Notably, only 10% of control health care professionals, most of whom were BLS or ACLS certified, successfully passed the combined assessment. These participants exhibited deficiencies in both knowledge performance and as team leaders during a simulated MCA event. This finding highlights the critical and urgent role of simulation-based blended learning, such as OBLS, in preparing for common maternal medical emergencies that may lead to MCA.

Our study found that knowledge retention was maintained in the intervention group for 6 months after OBLS education, whereas the control group did not demonstrate this retention. By 12 months, the intervention group exhibited a decrease in knowledge despite their self-efficacy scores remaining high. The control group had a decrease in knowledge by 6 months after the training despite self-efficacy scores remaining high through 12 months. The megacode scores were not assessed at the 6- or 12-month intervals, so it remains unclear whether the decrease in knowledge performance correlated with a decline in megacode team leader skills. Due to the low number of participants completing follow-up assessments, we cannot draw firm conclusions from this secondary analysis. However, prior studies^16,17,18^ of BLS and ACLS skills and knowledge retention have had similar findings. These findings on knowledge retention and the necessity of periodic refreshers provide valuable insights into the sustainability of the training’s impact.

### Limitations

We recognize important limitations in our trial. Our preliminary findings demonstrate an effect on participant knowledge; however, they do not yet show a reduction in maternal morbidity and mortality, which remains the ultimate translational goal of our educational programs. The study occurred in a highly controlled environment under the oversight of the course developers and used OBLS instructors with substantial experience in simulation and resuscitation education, potentially limiting generalizability to all settings in the US. We acknowledge 1 randomization error during the trial. A prehospital-advanced participant was randomized to a prehospital-basic control arm. Despite the error in randomization, this participant received prehospital OBLS education (same curricula for both prehospital advanced and basic groups) and the appropriate advanced assessments for their scope of work and remained in the prehospital group in the control arm for analysis. This minor randomization error is unlikely to significantly affect the analysis.

## Conclusions

This randomized clinical trial demonstrated the OBLS program’s effectiveness in enhancing health care professionals’ readiness to treat simulated maternal medical emergencies, including MCA. The significant improvements in cognitive performance, leadership during maternal codes, self-efficacy, and combined assessment pass rates among OBLS-trained participants highlight the program’s value across in-hospital and prehospital settings. However, innovative approaches to improve participant skills and knowledge retention for MCA skills are needed given the waning participant knowledge at 6 months. The current findings highlight the need for the broader implementation of resuscitation training to prepare health care professionals to respond to maternal medical emergencies.

## Notes

Supplement 1.
Trial Protocol

Supplement 2. **eFigure 1.** Prehospital Advanced Megacode Checklist **eFigure 2.** Prehospital Basic Megacode Checklist **eFigure 3.** In-Hospital Megacode Checklist **eFigure 4.** Orientation Checklist for In-Person Training

Supplement 3.
Data Sharing Statement
