# Supplementary material for: Obstetric Life Support Education for Maternal Cardiac Arrest: A Randomized Clinical Trial
Source: JAMA Netw Open. 2024 Nov 15;7(11):e2445295. doi: 10.1001/jamanetworkopen.2024.45295 (PMC11568455; doi:10.1001/jamanetworkopen.2024.45295)
Supplement: Supplement 1. — Trial Protocol [file jamanetwopen-e2445295-s001.pdf]

Supplemental Files

Trial Protocol and Statistical Analysis Plan

eMethods

Trial Protocol and Statistical Analysis Plan (SAP)

Evaluation of Obstetric Life Support (OBLS) Training Program for Responding to Maternal Medical Emergencies

Principal Investigator: Andrea Shields, MD, MS
Associate Professor of Obstetrics & Gynecology
University of Connecticut Health
Farmington, Connecticut

Protocol identification number: 22X-137

ClinicalTrials.gov identifier: NCT05355519

Author: Charles Minard, PhD
Statistician
Baylor College of Medicine
Houston, Texas

Version Draft 1.0

SIGNATURE PAGE

Principal Investigator ______________________
Andrea Shields (2024-01-31)

Author ______________________
Charles Minard (2024-01-31)

Table of Contents

1. Introduction 5

2. Study Objectives and Endpoints 5

3. Study Methods

3.1 General Study Design and Plan.. 5

3.2 Inclusion-Exclusion Criteria and General Study Population. 7

3.3 Randomization and Blinding.. 7

3.4 Study Assessments. 8

4. Sample size calculation 8

5. General Analysis Considerations

5.1 Timing of Analyses.. 8

5.2 Analysis Populations 9

5.2.1 Full Analysis Population (or Intention to Treat or Modified Intention to Treat)…........ 9

5.2.2 Per Protocol Population.. 9

5.2.3 Safety Population. 9

5.3. Covariates and Subgroups.. 9

5.4 Missing Data. 9

5.5 Multiple Testing. 9

6. Summary of Study Data

6.1 Subject Disposition.. 10

6.2 Derived variables.. 10

6.3 Protocol Deviations.. 10

6.4 Demographic and Baseline Variables. 10

7. Efficacy Analyses

7.1 Primary Efficacy Analysis. 11

7.2 Secondary Efficacy Analyses. 11

7.2.1 Secondary Analyses of Primary Efficacy Endpoint 11

7.2.2 Analyses of Secondary Endpoints. 11

8. Safety Analyses 12

9. Abbreviations 12

1 Introduction

1.1 Preface

Obstetric Life Support (OBLS) is a comprehensive training program designed to equip healthcare professionals with the necessary skills and knowledge to manage maternal medical emergencies and maternal cardiac arrest scenarios. This statistical analysis plan (SAP) will give more detailed descriptions of the endpoints in the study and the corresponding analyses.

1.2 Scope of the analyses

The study design is a prospective, single-blinded, randomized controlled trial with delayed intervention for the control group to evaluate the Obstetric Life Support (OBLS) education. The participants will be providers, nurses and GME learners currently certified in advanced cardiac life support (ACLS) or basic life support (BLS) and recruited from the anesthesiology, OB/GYN, emergency medicine, and critical care departments. Participants will be randomly allocated to receive OBLS education (Intervention arm) or no OBLS education (Control arm).

2 Study Objectives and Endpoints

2.1 Study Objectives

The objectives of this study are to develop and test the effectiveness of an educational program for improving knowledge and skills in managing maternal medical emergencies and maternal cardiac arrest among healthcare professionals.

2.2 Endpoints

2.2.1 Primary outcome measures

1. Cognitive test score

2.2.2. Secondary outcome measures

1. Confidence scores
2. Megacode scores
3. Combined assessment pass rate

3 Study Methods

3.1 General Study Design and Plan

This study will be a single-blind, randomized controlled trial with delayed intervention for controls. Participants will be randomized in a 1:1 ratio to Intervention (OBLS education) or

Control arm (delayed OBLS education). Participants will be randomized after enrollment. Evaluators will be blinded to the assigned study arm.

Participants assigned to the Intervention arm will receive cognitive didactic and interactive sessions and deliberate practice on a customized simulator. Cognitive and confidence evaluations are assessed at enrollment (Time 0), post-intervention (Time 1), 6-months post-enrollment (Time 2), and 12-months post-enrollment (Time 3). Megacode evaluations (technical and behavioral) will also be completed during Time 1.

Participants assigned to the Control arm will receive cognitive and confidence evaluations at enrollment (Time 1) but will not receive the educational intervention until 6-months post-enrollment (Time 2). Cognitive and confidence evaluations are then assessed post-intervention at the 6-month time point (Time 2) and 12-months post-enrollment (Time 3). Megacode evaluations are also completed during Time 1.

The primary outcome of interest is cognitive scores during Time 1. The superiority of the OBLS education will be assessed in the Intervention arm versus the Control arm during Time 1. Secondary outcomes include cognitive scores at other time points as well as confidence scores and Megacode scores.

The overall study design is summarized in Figure. At enrollment (Time 0), participants in the intervention arm will receive cognitive and self-efficacy assessments followed by receipt of the OBLS manual and prework 30-days prior to Time 1. The control arm will not receive any supplemental materials or instruction.

At Time 1, the intervention arm will receive OBLS in-person education with cognitive, self-efficacy, and Megacode assessment post-education. The control arm will receive baseline cognitive, self-efficacy assessments and Megacode assessment followed by receipt of the OBLS manual for prework 30-days prior to Time 2.

At Time 2 (6-months post-randomization), the intervention arm will be assessed for 6-month retention of cognitive and self-efficacy scores. The control arm will receive OBLS in-person education with cognitive, self-efficacy, and Megacode assessment post-education.

In Time 3 (12-months post-randomization), both study arms receive cognitive and self-efficacy assessments. This represents the 12-month knowledge retention for the intervention arm and 6-months for the control arm.

Figure. Flowchart of screening and inclusion process.

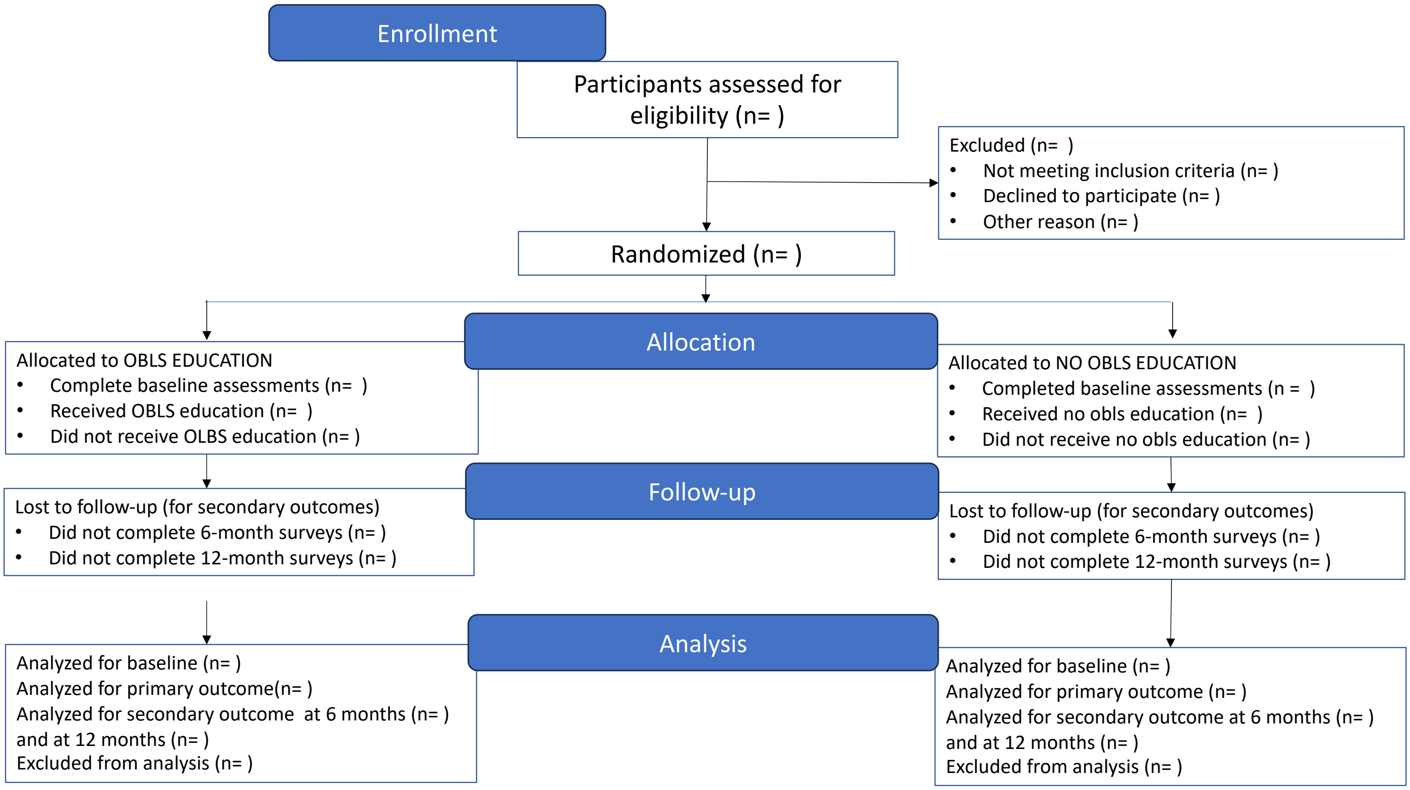

3.2 Inclusion-Exclusion Criteria and General Study Population

Study subjects will be English-speaking healthcare workers (18 years of age or greater) who work out-of-hospital (OH) or in-hospital (IH) contexts and care for reproductive-age women. For OH OBLS education, teams will have a maximum of four participants each consisting of two pairs of EMS healthcare professionals of varying levels, including law enforcement and firefighters. One participant must have at least five years or more in their current role. For IH OBLS education, teams will have a maximum of six participants consisting of at least one emergency medicine healthcare professional from Emergency Department (ED), Family Practice (FP), Intensive Care Unit (ICU), Surgery (Obstetrics [OB]); one anesthesiologist; one trainee of graduate medical education (GME) and/ one nurse from ED, FP, ICU/NICU, or OB/Labor & Delivery (L&D). Apart from the GME learners, at least one healthcare professional and one nurse must have at least five years of experience within their specialty. Exclusion criteria include anyone who meets eligibility criteria but who participated in our pilot validation trials and who do not consent to be randomized.

3.3 Randomization and Blinding

The study is a single-blinded RCT with two study arms. Subjects will be randomized to the

intervention or control in a 1:1 ratio using a permuted block design with random block sizes. Randomization will be stratified by hospital status (i.e., out-of-hospital versus in-hospital).

3.4 Study Assessments

Study assessments include the following validated instruments:

1. Cognitive test
   Developed and validated for participants from in- and out-of-hospital for online administration. Participants designated as out-of-hospital are further assigned to a basic versus advanced cognitive test based on their roles (e.g., EMTs that cannot administer medications or use advanced airways will be given the out-of-hospital, basic assessment). Each test item is equally weighted, and a final score is tabulated by computing the percent of items correct. Items with missing responses are assumed to be incorrect.

2. Megacode checklist
   Developed and validated for assessment of the team leader during a Megacode scenario of a maternal medical emergency by blinded evaluators. Participants from out-of-hospital contexts are further assigned to a basic versus advanced Megacode checklist test based on their roles (e.g., EMTs that cannot administer medications or use advanced airways will be given the out-of-hospital -basic Megacode checklist assessment). Each test item is on a Likert scale, and a final score is tabulated by adding up scores for each item and converting to a 100-point scale.

3. Self-efficacy assessment
   Developed and validated online assessment of confidence in four categories: clinical confidence, procedural confidence, knowledge confidence, and communication confidence. Each item is equally weighted, and a final score is tabulated by adding up all items and converting to a 100-point scale.

4 Sample Size Calculation

The primary outcome of interest is cognitive test scores during Time 1, and the primary hypothesis test will compare mean cognitive test scores during Time 1 between study arms using an independent, two-sample t-test. Preliminary data estimated the standard deviation of cognitive test scores to be about 12 points (Shields et al., 2023). A difference between means of 10-points is assumed to be clinically important. A sample size of 24 participants per group (N=48 total) provides 80% power to reject the null hypothesis that mean cognitive test scores are equal between study arms using an independent, two-sample t-tests assuming a common SD=12 and two-sided alpha = 0.05.

5 General Analysis Considerations

5.1 Timing of Analyses

Data will be analyzed after the trial is complete and will be defined as 3-months after Time 3 (12-months post-randomization).

5.2 Analysis Populations

5.2.1 Full Analysis Population (or Intention to Treat or Modified Intention to Treat)

Intention-to-treat (ITT)
All eligible, randomized participants. This will be the primary population for the analysis. Primary and secondary efficacy end points will be analyzed using intention-to-treat, with analyses including all eligible, randomized participants.

5.2.2 Per Protocol Population

All eligible, randomized participants who completed assessments at all time points. For a specific analysis, study subjects with missing data on any of the variables in the model will be excluded from the analysis. Analyses of this population is seen as a sensitivity analysis to investigate whether conclusions are sensitive to assumptions regarding the pattern of missing data.

5.2.3 Safety Population

We do not anticipate the need for safety population analysis due to low-risk intervention.

5.3. Covariates and Subgroups

We plan to analyze Megacode assessment outcomes based on the following subgroups: hospital-based versus out-of-hospital.

5.4 Missing Data

Cognitive test items that are missing will be scored as incorrect responses. Otherwise, scores for missing assessments (cognitive, self-efficacy, and megacode) will not be imputed. All available scores will be included in the primary analysis of the full population.

5.5 Multiple Testing

The primary outcome of interest is cognitive test scores. The primary hypothesis test will compare mean cognitive scores between treatment arms using an independent, two-sample t-test assuming homogenous variance and will be assessed at the two-sided 0.05 level. Secondary analysis will compare mean cognitive scores between arms across all time points using a general linear mixed model. If the arm-time interaction term is significant at the 0.05 level, then all pairwise comparisons will be assessed, and p-values will be adjusted for multiple comparisons using Tukey’s method.

Secondary outcomes include self-efficacy, megacode scores, and combined assessment pass rate (this includes meeting expert-derived minimum score for cognitive and megacode assessments). Self-efficacy scores will be analyzed like the primary outcome. Megacode are observed at a single time point and will be assessed at the two-sided 0.05 level. Combined assessment pass rate will compare the percent of participants who pass using Fisher’s exact test assessed at the two-sided 0.05 level.

6 Summary of Study Data

6.1 Subject Disposition

The number and proportion of screened, randomized, treated, and analyzed subjects will be provided. Where necessary, the CONSORT flow chart will be presented to describe the subject disposition in the statistical analysis report.

6.2 Derived variables

Cognitive scores will be computed as the percent of items correct and scores will range from 0% to 100%. Self-efficacy scores will be computed as the sum of item responses and converted to a 100-point scale. Scores will range from 0 to 100 points. Megacode scores will be computed as the percent of items correct and score will range from 0% to 100%. Participant age at enrollment will be computed as the integer age (rounded down) at the time of enrollment.

6.3 Protocol Deviations

For our trial, a protocol deviation is defined as a failure to adhere to the protocol such as the wrong intervention being administered, incorrect data being collected and documented, errors in applying inclusion/exclusion criteria or missed follow-up visits. Major deviations will include errors in randomization to OBLS versus no education, incorrect data being collected and documented, and errors in applying inclusion/exclusion criteria. Minor deviations include missed follow-up assessments. We will of summarize protocol deviations in the analysis (e.g., number and type of protocol deviations by intervention group or listing of all deviations) and provide details of whether the deviation is major or minor. If deviations occur, a sensitivity analyses will be conducted by removing patients with major deviations to assess impact on overall conclusions.

6.4 Demographic and Baseline Variables

Demographic/baseline variables will include age (years), gender identify (woman, man, non-binary), race/ethnicity (Asian/Black or African American, White, Hispanic/Latinx, Prefer not to answer), how many times participants have participated in a simulation exercise (< 5, 5-10, >10 and <20, and >20), if they have experience as a simulation instructor (yes/no),), if they are certified in BLS (currently certified, never certified or previously certified), if they are certified in ACLS (currently certified, never certified or previously certified), if they are certified in ATLS (currently certified, never certified or previously certified), if they are certified in PHTL (currently certified, never certified or previously certified), if they are certified in NRP (currently certified, never certified or previously certified), if they are certified as an BLS instructor (currently certified, never certified or previously certified), or if they are certified as an ACLS instructor (currently certified, never certified or previously certified). For normally distributed data comparing two means We will perform an independent, two sample test.

Baseline demographics and professional characteristics will be summarized by means with standard deviations, medians with minimum and maximum values, or frequencies with percentages. Summary statistics will be stratified by treatment arm and compared using

independent, two-sample t-tests, Wilcoxon rank sum test, chi-square test, or Fisher exact test as appropriate. Approximate normality will be assessed by quantile-quantile plots and Shapiro-Wilk test. Homogeneity of variances will be assessed by Levene's test. Table 1 presents an example table for summarizing and comparing baseline characteristics.

Table 1. Baseline participant characteristics

Characteristic	All (n=)	Intervention (n=)	Control (n=)	P-value
Age at enrollment in years				
N	N	N	N	
Mean (sd)	Mean (sd)	Mean (sd)	Mean (sd)	P
Gender, n (%)				
Woman	N (%)	N (%)	N (%)	P
Man	N (%)	N (%)	N (%)	
Non-binary	N (%)	N (%)	N (%)	
Race and Ethnicity, n (%)				
Asian	N (%)	N (%)	N (%)	P
Black or African American	N (%)	N (%)	N (%)	
White	N (%)	N (%)	N (%)	
Hispanic or Latino	N (%)	N (%)	N (%)	
Other (Prefer not to answer)	N (%)	N (%)	N (%)	
…	…	…	…	…

7 Efficacy Analyses

7.1 Primary Efficacy Analysis

The primary analysis will compare mean cognitive scores between treatment arms during Time 1 using an independent, two-sample t-test assuming equal variances as assessed at the two-sided alpha=0.05 level. Secondary analysis will use a general linear mixed model to compare mean cognitive scores between treatment arms across all time points. The model will include fixed effects for arm, time (i.e., Time 0, 1, 2, 3), and the arm-time interaction term. If the interaction term is significant at the 0.05 level, then all pairwise comparisons will be assessed by the model using linear contrasts and p-values will be adjusted for multiple comparisons using Tukey's method. Statistical significance will be assessed at the two-sided 0.05 level. The model will also estimate means with 95% confidence intervals by arm and time point. The matrix of correlated residuals will assume an unstructured format.

7.2 Secondary Efficacy Analyses

Secondary outcomes include self-efficacy scores, megacode scores, and combined assessment pass rate. Self- efficacy will be analyzed using a general linear mixed model similar to the secondary analysis of the primary outcome. Megacode scores will compare mean scores between treatment arms using

an independent, two-sample t-test. Combined assessment pass rates will estimate the frequency with percent of group passing the exam. Fisher exact test will compare percent passing between the two treatment arms. Statistical significance will be assessed at the 0.05 for all secondary outcomes.

8 Safety Analyses

Adverse events occurring during the OBLS education.

9 Abbreviations

ACLS Advanced Cardiac Life Support
ATLS Advanced Trauma Life Support
BLS Basic Life Support
CI Confidence Interval
IQR Interquartile Range
ITT Intention-to-Treat
NRP Neonatal Resuscitation Program
OBLS Obstetric Life Support
PHTLS Prehospital Trauma Life Support
PP Per Protocol
RCT Randomized Clinical Trial
SAP Statistical Analysis Plan

References

Shields, A. D., Vidosh, J., Thomson, B. A., Minard, C., Annis-Brayne, K., Kavanagh, L., Roth, C. K., Lutgendorf, M. A., Rahm, S. J., Becker, L. R., Mosesso, V. N., Schaeffer, B., Gresens, A., Epley, S., Wagner, R., Streitz, M. J., Bhalala, U. S., Melvin, L. M., Deering, S., & Nielsen, P. E. (2023). Validation of a Simulation-Based Resuscitation Curriculum for Maternal Cardiac Arrest
