# Supplementary material for: Obstetric Life Support Education for Maternal Cardiac Arrest: A Randomized Clinical Trial
Source: JAMA Netw Open. 2024 Nov 15;7(11):e2445295. doi: 10.1001/jamanetworkopen.2024.45295 (PMC11568455; doi:10.1001/jamanetworkopen.2024.45295)
Supplement: Supplement 2. — eFigure 1. Prehospital Advanced Megacode Checklist eFigure 2. Prehospital Basic Megacode Checklist eFigure 3. In-Hospital Megacode Checklist eFigure 4. Orientation Checklist for In-Person Training [file jamanetwopen-e2445295-s002.pdf]

Supplementary Online Content

Shields AD, Vidosh J, Minard C, et al. Obstetric Life Support education for maternal cardiac arrest: a randomized clinical trial. *JAMA Netw Open*. 2024;7(11):e2445295. doi:10.1001/jamanetworkopen.2024.45295

eFigure 1. Prehospital Advanced Megacode Checklist

eFigure 2. Prehospital Basic Megacode Checklist

eFigure 3. In-Hospital Megacode Checklist

eFigure 4. Orientation Checklist for In-Person Training

This supplementary material has been provided by the authors to give readers additional information about their work.

© 2024 Shields AD et al. *JAMA Network Open.*

eFigure 1. Prehospital Advanced Megacode checklist.

OBLS MEGACODE CHECKLIST - Out-of-Hospital Advanced

Name of Team Leader ______________________________________ **Scenario** ________________________

Directions: For each participant, use the scale provided and circle the number indicating the extent to which they completed the action. Items with * are critical components. *Participants must have no (0) critical fails in order to pass.*

Step	Assessment/Action	Done Correctly = 5<br>Done Poorly or Delayed = 3<br>Not Done = 0<br>N/A				
**RESUSCITATION**						
1	Performs primary survey	5	3	0	N/A	
2	Recognizes cardiac arrest*	5	3	0	N/A	
3	Calls for additional resources as needed	5	3	0	N/A	
4	Places patient on hard surface/safe location	5	3	0	N/A	
5	Initiates high-quality chest compressions*	5	3	0	N/A	1 min
6	Establishes and maintains effective ventilation (as staffing allows)*	5	3	0	N/A	
7	Ventilates at an appropriate rate	5	3	0	N/A	
8	Assesses possibility of pregnancy/assesses uterine fundus relationship to umbilicus	5	3	0	N/A	
9	Performs left uterine displacement*	5	3	0	N/A	
10	Anticipates potential need for resuscitative cesarean delivery	5	3	0	N/A	
11	Places defibrillation pads avoiding breast tissue	5	3	0	N/A	
12	Assesses cardiac rhythm immediately after pads placed	5	3	0	N/A	
13	Defibrillates if indicated, clears patient prior to shock*	5	3	0	N/A	2-5 mins
14	Inserts IV or IO above diaphragm	5	3	0	N/A	
15	Administers epinephrine					
	a. non-shockable: immediately	5	3	0	N/A	
	b. shockable: after two shocks	5	3	0	N/A	
16	Recognizes and treats reversible etiologies for maternal cardiac arrest, if applicable (e.g., Narcan for opioid overdose, etc.)	5	3	0	N/A	
17	Directs preparation for and transport of patient to most appropriate facility (OB Arrest Alert)*	5	3	0	N/A	
18	Applies ACCD correctly, if used	5	3	0	N/A	
	**A. Resuscitation SUBTOTAL (Items 1-18)**					
**POST-ROSC CARE**						
19	Verifies return of spontaneous circulation (ROSC)	5	3	0	N/A	
20	Evaluates AVPU	5	3	0	N/A	
21	Maintains airway and gives breaths every 6 seconds, as needed	5	3	0	N/A	POST-ROSC CARE
22	Maintains left uterine displacement	5	3	0	N/A	
23	Obtains and transmits 12-lead ECG	5	3	0	N/A	
24	Maintains vascular access and administers fluids	5	3	0	N/A	
25	Evaluates for causes	5	3	0	N/A	
	**B. Post-ROSC SUBTOTAL (Items 19-25)**					

© 2024 Shields AD et al. *JAMA Network Open.*

Step	Assessment/Action	Done Correctly = 5<br>Done Poorly or Delayed = 3<br>Not Done = 0<br>N/A				
**TEAM PERFORMANCE**						
26	Performs continuous LUD throughout resuscitation	5	3	0	N/A	TEAM PERF
27	Rotates personnel performing chest compressions and LUD every 2 min	5	3	0	N/A	
28	Minimizes chest compression interruptions (< 10 sec delay)	5	3	0	N/A	
29	Designated team member guides resuscitation with OBLS cognitive aid (ALIVE at 5)	5	3	0	N/A	
30	Keeps time of cardiac arrest and log of times/interventions	5	3	0	N/A	
31	Performs continuous high-quality CPR/OBLS until ROSC or higher level of care transfer achieved	5	3	0	N/A	
32	Uses BAACC TO LIFE for differential diagnosis	5	3	0	N/A	
	**C. Team Performance SUBTOTAL (Items 26-32)**					

Step	How well did the TEAM	Perfect = 5 Good = 4<br>Average = 3 Poor = 2<br>Unacceptable = 1				
**COMMUNICATION/TEAMWORK**						
33	Use SBAR to orient team members as they arrived?	5	4	3	2	1
34	Call for ADDITIONAL ASSISTANCE in a timely manner?	5	4	3	2	1
35	Utilize CLOSED-LOOP communication?	5	4	3	2	1
36	Maintain SITUATIONAL AWARENESS?	5	4	3	2	1
37	Utilize PATIENT FRIENDLY language and tone	5	4	3	2	1
**Step**	**Rate**					
38	OVERALL team communication	5	4	3	2	1
39	OVERALL team performance	5	4	3	2	1
	**D. Communication/Teamwork SUBTOTAL (Items 33-39)**					

**SCORING**		
		**Subtotals**
A	Resuscitation	
B	Post-ROSC	
C	Team Performance	
D	Total Communication/Teamwork	
E	Subtotal (add A + B + C + D)	
**F**	**Total points possible**	**200**
**G**	N/A adjustment (multiply # of N/A by 5)	
**H**	Adjusted total points (F minus G)	
**I**	Overall weighted score (divide E by H)	
**J**	Number of critical components* scored 0 (#1-21)	

SUMMARY

PASS = Overall score > 74% and no critical components scored 0.
Circle YES or NO.

**YES**	**NO**

Instructor Signature: ____________________

Instructor Name and Date: ____________________

Instructor potential? YES NO

© 2024 Shields AD et al. *JAMA Network Open.*

eFigure 2. Prehospital Basic Megacode checklist.

OBLS MEGACODE CHECKLIST - Out-of-Hospital BASIC

Name of Team Leader ______________________________________ **Scenario** ________________________

Directions: For each participant, use the scale provided and circle the number indicating the extent to which they completed the action. Items with * are critical components. *Participants must have no (0) critical fails in order to pass.*

Step	Assessment/Action	Done Correctly = 5<br>Done Poorly or Delayed = 3<br>Not Done = 0<br>N/A				
**RESUSCITATION**						
1	Performs primary survey	5	3	0	N/A	1 min
2	Recognizes cardiac arrest*	**5**	**3**	**0**	**N/A**	
3	Calls for additional resources as needed	5	3	0	N/A	
4	Places patient on hard surface/safe location	5	3	0	N/A	
5	Initiates high-quality chest compressions*	**5**	**3**	**0**	**N/A**	
6	Establishes and maintains effective ventilation (as staffing allows)*	**5**	**3**	**0**	**N/A**	
7	Ventilates at an appropriate rate and depth	5	3	0	N/A	
8	Assesses possibility of pregnancy/assesses uterine fundus relationship to umbilicus	5	3	0	N/A	
9	Performs LUD*	**5**	**3**	**0**	**N/A**	2-5 mins
10	Anticipates potential need for RCD	5	3	0	N/A	
11	Places defibrillation pads avoiding breast tissue	5	3	0	N/A	
12	Assesses cardiac rhythm with AED immediately after pads placed	5	3	0	N/A	
13	Defibrillates if indicated by AED, clears patient prior to shock*	**5**	**3**	**0**	**N/A**	
14	Directs preparation for and transport of patient to most appropriate facility (OB Arrest Alert)*	5	3	0	N/A	
15	Applies ACCD correctly, if used	5	3	0	N/A	
**A. Resuscitation SUBTOTAL (Items 1-15)**						
**POST-ROSC CARE**						
16	Verifies ROSC	5	3	0	N/A	POST-ROSC CARE
17	Evaluates AVPU scale (alert, verbal, pain, unresponsive)	5	3	0	N/A	
18	Maintains airway and gives breaths every 6 seconds, as needed	5	3	0	N/A	
19	Maintains LUD	5	3	0	N/A	
20	Obtains and transmits 12-lead ECG	5	3	0	N/A	
21	Maintains vascular access and administers fluids	5	3	0	N/A	
22	Evaluates for causes	5	3	0	N/A	
**B. Post-ROSC SUBTOTAL (Items 16-22)**						

© 2024 Shields AD et al. *JAMA Network Open.*

Step	Assessment/Action	Done Correctly = 5<br>Done Poorly or Delayed = 3<br>Not Done = 0<br>N/A				
**TEAM PERFORMANCE**						
23	Performs continuous LUD throughout resuscitation	5	3	0	N/A	TEAM PERF
24	Rotates personnel performing chest compressions and LUD every 2 min	5	3	0	N/A	
25	Minimizes chest compression interruptions (< 10 sec delay)	5	3	0	N/A	
26	Designated team member guides resuscitation with OBLS cognitive aid (ALIVE at 5)	5	3	0	N/A	
**C. Team Performance SUBTOTAL (Items 23-26)**						
**COMMUNICATION/TEAMWORK**						
**Step**	**How well did the TEAM**	**Perfect = 5 Good = 4<br>Average = 3 Poor = 2<br>Unacceptable = 1**				
27	Use SBAR to orient team members as they arrived?	5	4	3	2	1
28	Call for ADDITIONAL ASSISTANCE in a timely manner?	5	4	3	2	1
29	Utilize CLOSED-LOOP communication?	5	4	3	2	1
30	Maintain SITUATIONAL AWARENESS?	5	4	3	2	1
31	Utilize PATIENT FRIENDLY language and tone	5	4	3	2	1
**Step**	**Rate**					
32	OVERALL team communication	5	4	3	2	1
33	OVERALL team performance	5	4	3	2	1
**D. Communication/Teamwork SUBTOTAL (Items 27-33)**						

**SCORING**		
		**Subtotals**
A	Resuscitation	
B	Post-ROSC	
C	Team Performance	
D	Total Communication/Teamwork	
E	Subtotal (add A + B + C + D)	
**F**	**Total points possible**	**165**
**G**	N/A adjustment (multiply # of N/A by 5)	
**H**	Adjusted total points (F minus G)	
**I**	Overall weighted score (divide E by H)	
**J**	Number of critical components* scored 0 (#1-22)	

SUMMARY

PASS = Overall score > 74% and no critical components scored 0.
Circle YES or NO.

**YES**	**NO**

Instructor Signature:

Instructor Name and Date:

Instructor potential? **YES** **NO**

2021.06.15

© 2024 Shields AD et al. *JAMA Network Open.*

eFigure 3. In-hospital Megacode checklist.

OBLS MEGACODE CHECKLIST - In-Hospital

Name of Team Leader ______________________________ **Scenario** ____________________

Directions: For each participant, use the scale provided and circle the number indicating the extent to which they completed the action. Items with * are critical components. *Participants must have no (0) critical fails in order to pass.*

Step	Assessment/Action	Done Correctly = 5<br>Done Poorly or Delayed = 3<br>Not Done = 0<br>N/A				
	**RESUSCITATION**					
1	Recognizes unstable vital signs in pregnant patient	5	3	0	N/A	**1 min**
2	Recognizes cardiac arrest*	5	3	0	N/A	
3	Activates MCAT	5	3	0	N/A	
4	Positions patient flat and places backboard	5	3	0	N/A	
5	Initiates high-quality chest compressions*	5	3	0	N/A	
6	Establishes and maintains effective ventilation*	5	3	0	N/A	
7	Ventilates at appropriate rate	5	3	0	N/A	
8	Assesses possibility of pregnancy/assesses uterine fundus relationship to umbilicus	5	3	0	N/A	
9	Performs and maintains LUD throughout resuscitation*	5	3	0	N/A	
10	Gathers equipment for RCD	5	3	0	N/A	**2-5 mins**
11	Places defibrillation pads avoiding breast tissue	5	3	0	N/A	
12	Assesses cardiac rhythm immediately after placing pads*	5	3	0	N/A	
13	Removes fetal monitors	5	3	0	N/A	
14	Defibrillates if indicated, clears patient before shock*	5	3	0	N/A	
15	Inserts IV or IO above diaphragm	5	3	0	N/A	
16	Administers epinephrine:<br>a. non-shockable: immediately	5	3	0	N/A	
	b. shockable: after two shocks	5	3	0	N/A	
17	Assesses cardiac rhythm and performs pulse check every 2 minutes*	5	3	0	N/A	
18	Recognizes and treats reversible etiologies for MCA, if applicable (e.g., calcium gluconate for magnesium toxicity, Narcan for opioid overdose)	5	3	0	N/A	
19	Completes RCD by 5 min at site of arrest*	5	3	0	N/A	
20	Maintains chest compression effectiveness throughout, minimizes chest compression interruptions (< 10 sec delay)	5	3	0	N/A	**5+ mins**
21	Timely notification of contingency teams<br>(i.e., ECPR, MAST, vascular surgeon)	5	3	0	N/A	
22	Verifies ROSC	5	3	0	N/A	
	**A. Resuscitation SUBTOTAL (Items 1-21)**					
	**POST-ROSC CARE**					
23	Discusses post-arrest care elements following ROSC to establish next steps in management	5	3	0	N/A	POST-ROSC CARE
24	Secures airway and gives breaths every 6 seconds, as needed	5	3	0	N/A	
25	Maintains LUD, if pregnant	5	3	0	N/A	
26	Maintains vascular access and administers fluids	5	3	0	N/A	
27	Uses BAACC TO LIFE to consider potential etiology(ies)	5	3	0	N/A	
28	Manages wounds (closure, antibiotics)	5	3	0	N/A	
29	Orders head CT prior to transfer to ICU, if indicated	5	3	0	N/A	
	**B. Post-ROSC Care SUBTOTAL (Items 22-28)**					

© 2024 Shields AD et al. *JAMA Network Open.*

TEAM LEADER PERFORMANCE						
Step	Assessment/Action	Done Correctly = 5<br>Done Poorly or Delayed = 3<br>Not Done = 0<br>N/A				
30	Effectively guides resuscitation, focusing on high-quality chest compressions and continuous LUD	5	3	0	N/A	LEAD PERF
31	Effectively delineates responsibilities to team members	5	3	0	N/A	
32	Ensures team member keeps time of cardiac arrest and logs times/interventions	5	3	0	N/A	
33	Ensures contingency teams are present and working together effectively	5	3	0	N/A	
34	Recommends debrief w/ OBLS debrief tool	5	3	0	N/A	
	**C. Team Leader Performance SUBTOTAL (Items 29-33)**					

COMMUNICATION/TEAMWORK						
Step	How well did the TEAM	Perfect = 5 Good = 4<br>Average = 3 Poor = 2<br>Unacceptable = 1				
35	Use SBAR to orient team members as they arrived?	5	4	3	2	1
36	Call for ADDITIONAL ASSISTANCE in a timely manner?	5	4	3	2	1
37	Utilize CLOSED-LOOP communication?	5	4	3	2	1
38	Maintain SITUATIONAL AWARENESS?	5	4	3	2	1
39	Utilize PATIENT FRIENDLY language and tone	5	4	3	2	1
	**Rate**					
40	OVERALL team communication	5	4	3	2	1
41	OVERALL team performance	5	4	3	2	1
	**D. Communication/Teamwork SUBTOTAL (Items 34-40)**					

SCORING		
		Subtotals
A	Resuscitation	
B	Post-ROSC	
C	Team Performance	
D	Total Communication/Teamwork	
E	Subtotal (add A + B + C + D)	
**F**	**Total points possible**	**210**
**G**	N/A adjustment (# of N/A__________ multiplied by 5)	
**H**	Adjusted total points (F minus G)	
**I**	Overall weighted score (divide E by H)	
**J**	Number of critical components* scored 0 (#1-22)	

SUMMARY

PASS = Overall score > 74% and no critical components scored 0.
Circle YES or NO.

**YES**	**NO**

Instructor Signature:

____

Instructor Name and Date:

____

Instructor potential? YES NO

2024.02.08

© 2024 Shields AD et al. *JAMA Network Open.*

eFigure 4. Orientation checklist for In-Person Training.

RCT IH MORNING
Pre-RCDP Orientation Checklist

Checklist Purpose: To guide instructors as they introduce the OBLS in-person course.
Timing: In the morning, before starting the rapid-cycle deliberate practice training scenarios.

❑	**Introduce** yourself
❑	**Invite** participants to introduce themselves and current profession
❑	**Emphasize** that the simulated environment allows for mistakes
❑	**Set the stage**<br>• Remind participants that everyone is capable, smart, and desires to learn.<br>• State that you will interrupt them and give specific feedback about what you see.<br>• Normalize mistakes and let them know that they'll repeat tasks.<br>○ "You will make mistakes. If you were teaching and I were the student, I would be making mistakes."<br>**Ask participants to**<br>• treat the scenario as real as possible<br>• take care of patients within their current scope of practice (i.e., RN does not do RCD)<br>• participate as fully as possible<br>• remain focused on the training
❑	**Introduce rapid-cycle deliberate practice**<br>• Real-time corrections to create new skills through repetition and practice<br>• Maximizes learning by reinforcing learning in real-time, while emphasizing teamwork<br>• "I'll be calling timeouts to provide immediate feedback and correct the mistake. Then, we'll rewind 30 seconds to repeat the scenario so you can practice the appropriate action."<br>• Request participants to ask questions and call time outs when something isn't clear
❑	**Introduce the manikin's features and technology, as applicable**<br>• Recognize the limitations of manikin<br>○ "Skin doesn't change color or feel or look real, and she has no extremities"<br>• Demonstrate left uterine displacement (LUD) from manikin's left and show indicator color change<br>○ Invite participants to briefly practice LUD<br>• Note chest compressions monitor on iPad<br>• Demonstrate resuscitative cesarean delivery and how to place backboard<br>• Indicate defibrillator pad placement avoiding breast tissue<br>• Note intubatable airway, demonstrate head-tilt/chin lift, jaw thrust, capnography<br>• Demonstrate rotation of compressor and LUD participants
❑	**Instruct** participants to do everything at/to the manikin (e.g., ask questions, physical exam, procedures)
❑	**Orient participants to**<br>• resuscitative cesarean delivery kit<br>• hemorrhage cart<br>• simulated blood products<br>• crash cart with simulated medications

6/6/2023

© 2024 Shields AD et al. *JAMA Network Open.*

	○ participants should locate meds and place beside manikin; do not need to draw them up<br>• airway equipment<br>• defibrillator and pads<br>○ participants should place pads on manikin and turn on and charge machine; instructors will show rhythm strips
❑	**Introduce participant roles**<br>• 6 participants<br>1. Team lead<br>2. Compressor<br>3. LUD<br>4. Crash cart<br>5. Airway<br>6. Recorder*<br>*if <6 participants, combine crash cart and recorder<br>• Participants activate MCAT, provide SBAR, and assist team leader
❑	**Introduce instructor roles**<br>• will pause training scenarios to provide rapid cycle deliberate practice<br>• will end the scenario and guide participants<br>• during megacode<br>○ One instructor leads the simulation, acts as the patient, ensures proper case flow, and both grade the team leader with the megacode checklist
❑	**Introduce scenario flow**<br>• Three training scenarios with timeouts to correct action<br>○ Prearrest<br>○ arrest<br>○ post-ROSC using post-ROSC checklist<br>○ debrief using megacode checklist<br>○ sharing difficult news<br>• Each training scenario will be different and each megacode different from the training scenarios<br>• Each participant will be a team lead in the morning and afternoon<br>• If 6 participants, each morning scenario will have 2 team leads who switch in scenario middle<br>• Instructors will<br>○ give 1st participant HPI/initial scenario<br>○ stop and correct mistakes<br>• Scenarios will progress into arrest and end in ROSC once the team completes various steps

© 2024 Shields AD et al. *JAMA Network Open.*
