# Supplementary material for: Obstetric Life Support Education for Maternal Cardiac Arrest: A Randomized Clinical Trial
Source: JAMA Netw Open. 2024 Nov 15;7(11):e2445295. doi: 10.1001/jamanetworkopen.2024.45295 (PMC11568455; doi:10.1001/jamanetworkopen.2024.45295)
Supplement: Supplement 3. — Data Sharing Statement [file jamanetwopen-e2445295-s003.pdf]

Data Sharing Statement

Shields. Obstetric Life Support Education for Maternal Cardiac Arrest. *JAMA Netw Open*. Published November 15, 2024. doi:10.1001/jamanetworkopen.2024.45295

Data
Additional Information: Clinicaltrials.gov, https://clinicaltrials.gov/study/NCT05355519, NCT05355519
Data available: Yes
Data types: Deidentified participant data
How to access data: The steering committee will evaluate all requests. Requests should be sent to ashields@uchc.edu
When available: With publication

Supporting Documents
Document types: Statistical/analytic code, Informed consent form
How to access documents: R custom script. The steering committee will evaluate all requests. Requests should be sent to ashields@uchc.edu
When available: With publication

Additional Information
Who can access the data: researchers whose proposed use of the data has been approved
Types of analyses: for a specified purpose
Mechanisms of data availability: without investigator support, after approval of a proposal, or with a signed data access agreement).
Any additional restrictions: N/A
